# Percutaneous transhepatic biliary drainage in patients with cholestasis following liver transplantation

**DOI:** 10.1007/s00261-024-04657-2

**Published:** 2024-11-05

**Authors:** Thorben Pape, Tabea von Garrel, Anna M. Hunkemöller, Bahar Nalbant, Florian W. R. Vondran, Nicolas Richter, Benjamin Heidrich, Andrea Schneider, Richard Taubert, Thomas von Hahn, Heiner Wedemeyer, Benjamin Seeliger, Henrike Lenzen, Klaus Stahl

**Affiliations:** 1 Department of Respiratory Medicine and Infectious Diseases, Hannover Medical School, Hannover, Germany; 2 Department of Gastroenterology, Hepatology, Infectious Diseases and Endocrinology, Hannover Medical School, Hannover, Germany; 3 Present Address: Department of General, Visceral, Pediatric and Transplant Surgery, University Hospital RWTH Aachen, Aachen, Germany; 4 Department of General, Visceral and Transplant Surgery, Hannover Medical School, Hannover, Germany; 5 Present Address: Department of Gastroenterology and Interventional Endoscopy, Hospital Hamburg-Barmbek, Hamburg, Germany; 6 Biomedical Research in End-Stage and Obstructive Lung Disease Hannover, German Center for Lung Research, Hannover, Germany

**Keywords:** Liver transplantation, PTBD, Cholestasis, Cholangitis, Biliary strictures

## Abstract

**Purpose:**

Biliary strictures are among the most common complications following liver transplantation (LT). If endoscopic retrograde cholangiography fails, percutaneous transhepatic biliary drainage (PTBD) may serve as an alternative approach. Description of clinical important short- and long-term outcomes as well as outcome prediction following PTBD after LT are scarce.

**Methods:**

We analyzed outcomes of 56 liver-transplanted adults with biliary complications receiving a PTBD. We described the safety and longitudinal laboratory changes. We analyzed as endpoints, incidence of biliary complications, need for surgical biliary revision/re-LT and overall-survival at 12- and 60-months. We used simple comparison tests accordingly and performed competing risk analysis and multivariate competing risk regression as well as log-rank test and cox proportional hazard regression for further analysis.

**Results:**

PTBD procedures had a high technical success rate (98%) and tolerable safety profile. Multiple laboratory indicators improved during follow-up (37 patients with complete biochemical follow-up). Incidence of subsequent biliary complications was highly dependent on the nature of present biliary strictures (Anastomotic stricture (AS): adjusted SHR: 0.26, 95% CI: 0.09–0.78, p = 0.016). Need for surgical biliary revision/re-LT remained below 15%. 12-month survival was significantly better, if drainage into the small intestine was achieved at first attempt compared to completely external drainage (internal: 92.9 vs. external: 67.9%, p = 0.018). Patients with AS had a numerically higher long-term-survival and higher C-reactive-protein (CRP) and lower body-mass-index (BMI) at baseline were significantly associated with inferior short- and long-term-survival.

**Conclusion:**

PTBD for biliary complications following LT had a high technical success and a tolerable safety profile. Incidence of subsequent biliary complications was highly dependent on the nature of biliary strictures and increased mortality was found in patients with higher CRP, lower BMI and failure of initial PTBD internalization.

**Graphical abstract:**

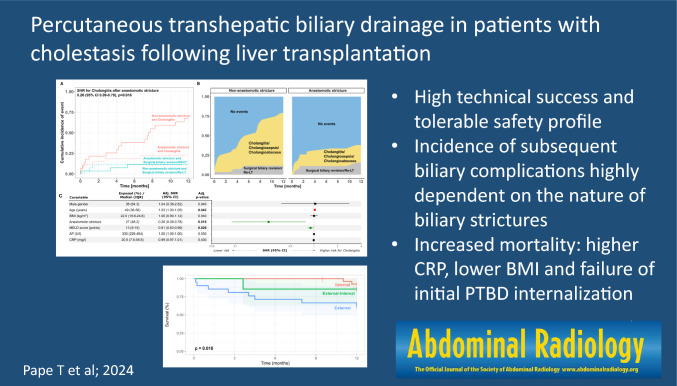

**Supplementary Information:**

The online version contains supplementary material available at 10.1007/s00261-024-04657-2.

## Background

Biliary strictures are among the most common complications following liver transplantation (LT) and may occur in up to 20% of patients [1]. Both anastomotic- (AS) as well as non-anastomotic biliary strictures (NAS) cause cholestasis and may then lead to biliary complications such as cholangitis and cholangiosepsis, formation of biliary abscesses, development of biliary cirrhosis and ultimately, graft loss resulting in re-LT or death [2, 3]. The disparity between the need and the availability of donor organs further emphasizes the need for early and effective treatment of these complications to prevent graft loss after successful LT [3].

The standard approach to access the biliary system and to resolve biliary strictures is by endoscopic retrograde cholangioscopy (ERC) [3, 4]. However, if ERC fails or is not feasible due to the presence of a primary biliodigestive anastomosis (BDA), percutaneous transhepatic biliary drainage (PTBD) may serve as an alternative approach to treat biliary strictures following LT [5–7].

While multiple potential risk factors for the development of biliary strictures following LT have been explored [8–10], there still remains a major paucity of data concerning clinical important short- and long-term outcomes as well as outcome prediction following PTBD treatment after LT [7, 11]. These are, however, crucial to underline the clinical value of PTBDs in this special patient cohort.

Therefore, in this retrospective, single-center study from a large liver transplant center, we aimed to systematically describe the outcome of LT recipients who had biliary complications post-LT requiring PTBD.

## Materials and methods

### Screening and inclusion into the study

**Fig. 1:**
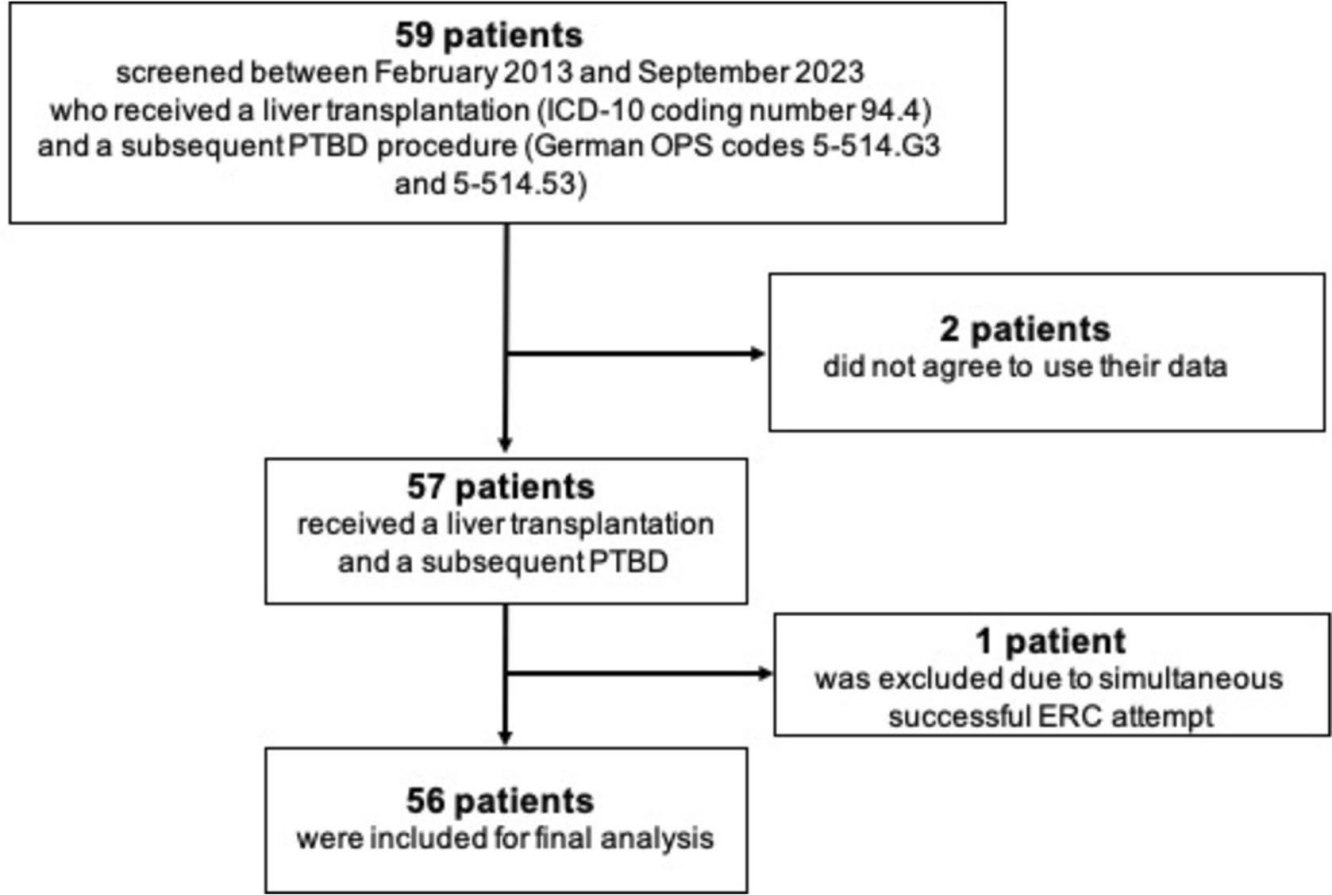
*Study inclusion.* 59 LT adults received a PTBD between February 2013 and January 2023. Two patients did not agree to use their data and one patient was excluded due to a successful simultaneous ERC attempt. Therefore, 56 patients were included into the study for final analysis (intention-to-treat analysis). *ERC* endoscopic retrograde cholangioscopy*, LT* Liver transplantation*, PTBD* Percutaneous transhepatic biliary drainage

This is a retrospective, single-center study investigating the outcome of 56 liver-transplanted adults (ICD-10 coding number 94.4) with cholestasis receiving a subsequent PTBD (German OPS codes 5–514.G3 and 5–514.53). Initially, 59 LT adults receiving a PTBD at a tertiary care hospital between February 2013 and January 2023 were included. Two patients were excluded as they did not agree to use their data and one patient was excluded due to a successful simultaneous endoscopic retrograde cholangioscopy (ERC) attempt (Fig. 1). PTBD was attempted in 56 consecutive patients and successfully placed in 55 (98%). An intention-to-treat analysis was performed. The study was performed according to the ethical standards that were laid down in the Declaration of Helsinki 1964 and its later amendments. Accordingly, informed consent was obtained from patients and/or legal representatives. The local institutional review board (Nr. 10941_BO_K_2023) approved the study protocol.

### Transhepatic biliary drainage [percutaneous transhepatic biliary drainage (PTBD)] insertion and further handling

PTBD was indicated when persisting cholestasis was present based on biochemical parameters and imaging by transabdominal sonography or magnetic resonance imaging (MRI). PTBD insertion was performed following single-shot antibiotic prophylaxis and under general anesthesia. First, a prominent intrahepatic biliary branch was located and punctured under sonographic guidance with a 22 G needle. Bile was aspirated. If contrast visualization confirmed the (dilated) bile ducts up to the anastomosis, initial biliary cannulation was performed using a 0.018-inch platin wire (Cook® Medical, Limerick, Ireland) and 4–6 F catheter sheath (Cook® Medical, Limerick, Ireland). Then the wire was changed to a 0.035-inch standard wire (Terumo, Eschborn, Germany) and intubation of the central biliary system and the biliary anastomosis was attempted under fluoroscopic guidance. Contrast imaging then confirmed the correct positioning into the small intestine. Further bougienage with different size bougies up to 9 F was then performed over the guide wire. Eventually, a 6–8 F “Munich” biliary drainage (Peter Pflugbeil GmbH, München, Germany) was placed with the distal end reaching the small intestine. If a small intestinal loop was not reached, exclusive drainage to the outside was used instead. PTBD was then routinely exchanged every three months over a wire and under fluoroscopic guidance. If the targeted biliary stricture persisted, PTBD size was increased up to 12–16 F with intervention via bougienage or balloon dilatation as needed. PTBD was removed if parameters of cholestasis were regressive and no signs of persistent biliary stenosis could be visualized on imaging any more. If cholestasis was progressive despite PTBD or biliary complications such as cholangitis or cholangiosepsis occurred, the further strategy was discussed in an interdisciplinary team consisting of transplant surgeons and interventional gastroenterologists to guide further approaches towards surgical biliary revision or ultimate re-LT.

### Outcome parameters

We aimed to describe outcomes of LT adults undergoing PTBD due to biliary complications. Procedural characteristics including technical success rate, drainage strategy (extern vs. intern), maximal PTBD size and number of PTBD exchanges, rate of successful stricture resolution, PTBD demission and reinsertion as well as safety of the intervention were recorded. Changes in laboratory indicators of cholestasis (i.e. gamma glutamyl transferase (GGT) and alkaline phosphatase (AP)), graft function represented by the “model for the end stage of liver disease” (MELD) score and inflammation (C-reactive protein (CRP)) were analyzed between initial PTBD placement (baseline) and follow-up (six and twelve months following PTBD insertion) for all patients with a complete follow-up (n = 37).

As co-primary endpoints, incidence of biliary complications (cholangitis, cholangiosepsis, biliary abscess) and need for surgical biliary revision or re-transplantation was analyzed at 12 and 60 months following initial PTBD placement. Cholangitis was defined by presence of the Tokyo criteria [12]. Cholangiosepsis was defined as acute cholangitis with co-existence of sepsis, as defined by the sepsis-3 consensus [13].

As key secondary endpoint, overall survival was recorded at 12 (and 60 months). Finally, potential baseline predictors of both the primary- and key secondary outcomes were identified using multivariate competing risk- as well as cox-proportional hazard regression modelling, respectively.

### Data collection

Data were collected using electronic medical records including the patient data monitoring system (PDMS) SAP and ALIDA. MELD scores were calculated according to the description by Kamath PS et al. [14] (including bilirubin, INR and creatinine). All personal patient data were pseudo-anonymized before further analysis.

### Statistical analysis

GraphPad Prism (Version 9.0, GraphPad Software, La Jolla, CA) and the R environment for statistical computing (Version 4.1.2, R Foundation for Statistical Computing, Vienna, Austria) were used for data analysis and graph generation. Categorical variables are shown as numbers (n) and percentages (%). Unless indicated otherwise, continuous variables are shown as median and 25%–75% quartiles. Variables were checked for normal distribution using the D’Agostino-Pearson omnibus normality test and the Shapiro–Wilk normality test. For comparisons, Mann–Whitney U test, Wilcoxon matched—pairs signed rank test, two-sided paired t-test, one-way ANOVA, Friedmann-test and Kruskal–Wallis test were used accordingly. The co-primary endpoints, occurrence of cholangitis/cholangiosepsis/biliary abscess, need for surgical biliary revision and liver re-transplantation, were analyzed and visualized by competing risk analysis and multivariate competing risk regression (R packages *cmprsk* and *finalfit*). A narrowed set of covariables was used for the multivariate competing risk regression: gender, age, BMI, anastomotic stricture, as well as MELD score, AP, and CRP at PTBD admission. The hazard ratio (HR) for occurrence of cholangitis/cholangiosepsis/biliary abscess between anastomotic and non-anastomotic stricture is stated as the subdistribution hazard ratio (sHR). Survival was visualized by Kaplan–Meier graphs and analyzed by Log-rank test as well as Cox proportional hazard regression (R packages *survival* and *survminer*). In the multivariable model, gender, age, BMI, anastomotic stricture, as well as MELD score, AP, and CRP at PTBD admission were selected as fixed covariables. All reported p-values are two-sided unless indicated otherwise; p-values < 0.05 were considered statistically significant.

## Results

### Cohort characterization

A PTBD was attempted in a total of 56 LT adults between February 2013 and January 2023. Demographic and clinical characteristics at the time of initial PTBD insertion (= baseline) are demonstrated in Table 1. Median (Interquartile Range (IQR)) age was 49 (36 – 58) years with 36 (64%) male patients. The main reason for LT was primary sclerosing cholangitis (PSC) (41%), followed by hepatitis B or C infection (9%) and autoimmune hepatitis (7%). 14 patients had a variety of rare causes for LT, e.g. Budd-Chiari-syndrome or secondary sclerosing cholangitis (SSC), and were categorized as others. Almost all patients received a full organ transplant (86%) from a deceased donor (98%). A BDA was established in 47 patients (86%). Primary PTBD without previous ERC attempt was performed in 23 (41%) patients. A NAS was the most common indication for PTBD (55%), followed by a AS (48%). Biliary abscesses, cholelithiasis, PSC relapse and anastomotic leakage were concurring further diagnoses in addition to NAS and AS.

**Table 1:** Demographic and clinical characteristics at first PTBD insertion

Category	Median (IQR)/No (%)
	(n = 56)
Age—years	49 (36–58)
Sex—no (%)	
Male	36 (64.3)
Female	20 (35.7)
Height—m	1.75 (1.67–1.80)
Weight—kg	68.0 (55.3–79.8)
BMI—kg/m^2^	22.0 (19.6–24.8)
Indication for LT—no (%)	
PSC	23 (41.1)
Biliary atresia	3 (5.4)
Acute liver failure	2 (3.6)
Hepatitis B or C infection	5 (8.9)
Autoimmune hepatitis	4 (7.1)
HCC	3 (5.4)
Alpha-1 antitrypsin deficiency	2 (3.6)
Others	14 (25.0)
Living liver donor—no (%)	1 (1.8)
Split-LT—no (%)	8 (14.3)
BDA—no (%)	47 (85.5)
Primary PTBD attempt—no (%)	23 (41.1)
Reason for PTBD insertion—no (%)	
Anastomotic stricture	27 (48.2)
Non-anastomotic stricture	31 (55.4)
Biliary abscess	11 (19.6)
Cholelithiasis	5 (8.9)
Anastomotic Insufficiency	2 (3.6)
Biliocutaneous fistula	1 (1.8)
PSC relapse	5 (10.7)
Cholangiocarcinoma	1 (1.8)
Complications prior to PTBD—no (%)	
Cholangitis—no (%)	42 (76.4)
Cholangiosepsis—no (%)	15 (26.8)
Cholangioabscess—no (%)	15 (26.8)
Surgical biliary revision—no (%)	12 (21.4)
AP—U/l	330 (229–494)
GGT—U/l	337 (182–713)
Bilirubin total—µmol/l	28 (14–77)
MELD	13 (9–19)
CRP—mg/l	20.5 (7.6–55.6)

*AP* alkaline phosphatase, *BDA* biliodigestive anastomosis, *BMI* body mass index, *CCA* cholangiocarcinoma, *CRP* C-reactive protein, *GGT* gamma glutamyl transferase, *HCC* hepatocellular carcinoma, *IQR* interquartile range, *ITBL* ischemic type biliary lesions, *LT* liver transplantation, *MELD* model of end stage liver disease, *PSC* primary sclerosing cholangitis, *PTBD* percutaneous transhepatic biliary drainage

Demographic and clinical characteristics of the whole patient cohort (n = 56) who received a PTBD at baseline (PTBD insertion, intention-to-treat). Multiple mentions are possible as indication for LT and laboratory indicators at baseline are shown for the whole patient cohort (n = 56). Values are presented as median (25% to 75% IQR) or if categorical as numbers and percentages.

### Procedural characteristics and safety of PTBD

Initial PTBD insertion was successful in 55 patients (98%) (Table 2). PTBD was attempted to be directed with the distal tip into the small intestine in all patients. In 28 (50%) of patients the drainage could be placed into the small intestine at the time of initial PTBD insertion (internal). In the remaining cases an external drainage was placed initially and was either redirected to the small intestine throughout the subsequent interventions (external-internal) (12.5%) or kept as an exclusive external drainage (37.5%). Overall, an internal drainage was achieved either initially or through subsequent PTBD exchanges in 62.5% of patients (Table 2). Of note, in four patients two simultaneous PTBDs were established. In three of these patients a PTBD was placed in both liver lobes simultaneously. For simplification in patients with two drainages, only the first PTBD placement, which was exclusively external in all patients (three with additional external-internal and one with additional internal through following procedures), was taken into account. External-internal and external PTBDs were categorized as initially external PTBDs for further analysis unless otherwise specified. The median size of the first PTBD was 8 (8–9) French. Biliary dilatations in addition to push dilatation by PTBD exchanges, were performed by bougienage in 48 patients (86%) and subsequently, strictures were resolved in 63% of patients.

**Table 2:** Procedural and safety characteristics

Category	Median (IQR)/No (%)
	(n = 56)
Technical success—no (%)	55 (98.2)
Location of PTBD—no (%)	
PTBD right liver lobe	41 (73.2)
PTBD left liver lobe	18 (32.1)
PTBD both liver lobes	3 (5.4)
Drainage strategy—no (%)	
External drainage only	21 (37.5)
Initial External drainage only, internal drainage later achieved	7 (12.5)
Internal drainage at first attempt	28 (50.0)
Size of first PTBD—Fr	8 (8–9)
Number of total PTBD changes	4 (1–9)
Maximum size of PTBD—Fr	16 (10–16)
Cholangioscopy—no (%)	3 (5.4)
Dilatation—no (%)	
Bougie	48 (85.7)
Balloon	1 (1.8)
Strictures resolved—no (%)	35 (62.5)
PTBD dislocated—no (%)	17 (30.4)
PTBD demission—no (%)	31 (55.4)
Time to PTBD demission	12 (4–34)
PTBD reinsertion—no (%)	13 (23.2)
Procedure related complications—no (%)	13 (23.2)
Bleeding	6 (10.7)
Cholangitis	2 (3.6)
Cholangiosepsis	3 (5.4)
Other	2 (3.6)

*PTBD* percutaneous transhepatic biliary drainage, *IQR* interquartile range

Procedure related complications were defined as complications directly during or after PTBD admission and were noted in a total of 13 patients (23%). The most common procedural related complication was bleeding in six (11%), followed by cholangiosepsis in three (5%) and cholangitis in two (4%) patients (Table 2).

Procedural characteristics and procedural-related complications (defined as directly related to the PTBD procedure) for the PTBD insertion are shown. Of note, in four patients two simultaneous PTBDs were established. In three of these patients a PTBD was placed in both liver lobes simultaneously. For simplification in patients with two drainages only the first PTBD is taken into account (in all four patients extern). External-internal and external PTBDs were categorized as initially external PTBDs for further analysis unless otherwise specified. Values are presented as median (25% to 75% IQR) or if categorical as numbers and percentages.

Median follow-up time was 39.8 (15.0–67.2) months. PTBD could be removed in 31 patients (55%) overall after a median of 4 (1–9) PTBD changes and a median time of 12 (4–34) months (Table 2, Supp. Fig. 1A). Interestingly, PTBDs placed in patients with AS could be removed numerically more often than in those placed in NAS (Supp. Fig. 1B). PTBDs needed to be reinserted in 13 patients (23%), respectively (Table 2, Supp. Fig. 1C) without any differences between AS and NAS biliary strictures (Supp. Fig. 1D).

### Longitudinal changes in laboratory indicators of cholestasis, graft function and inflammation following PTBD

**Fig. 2:**
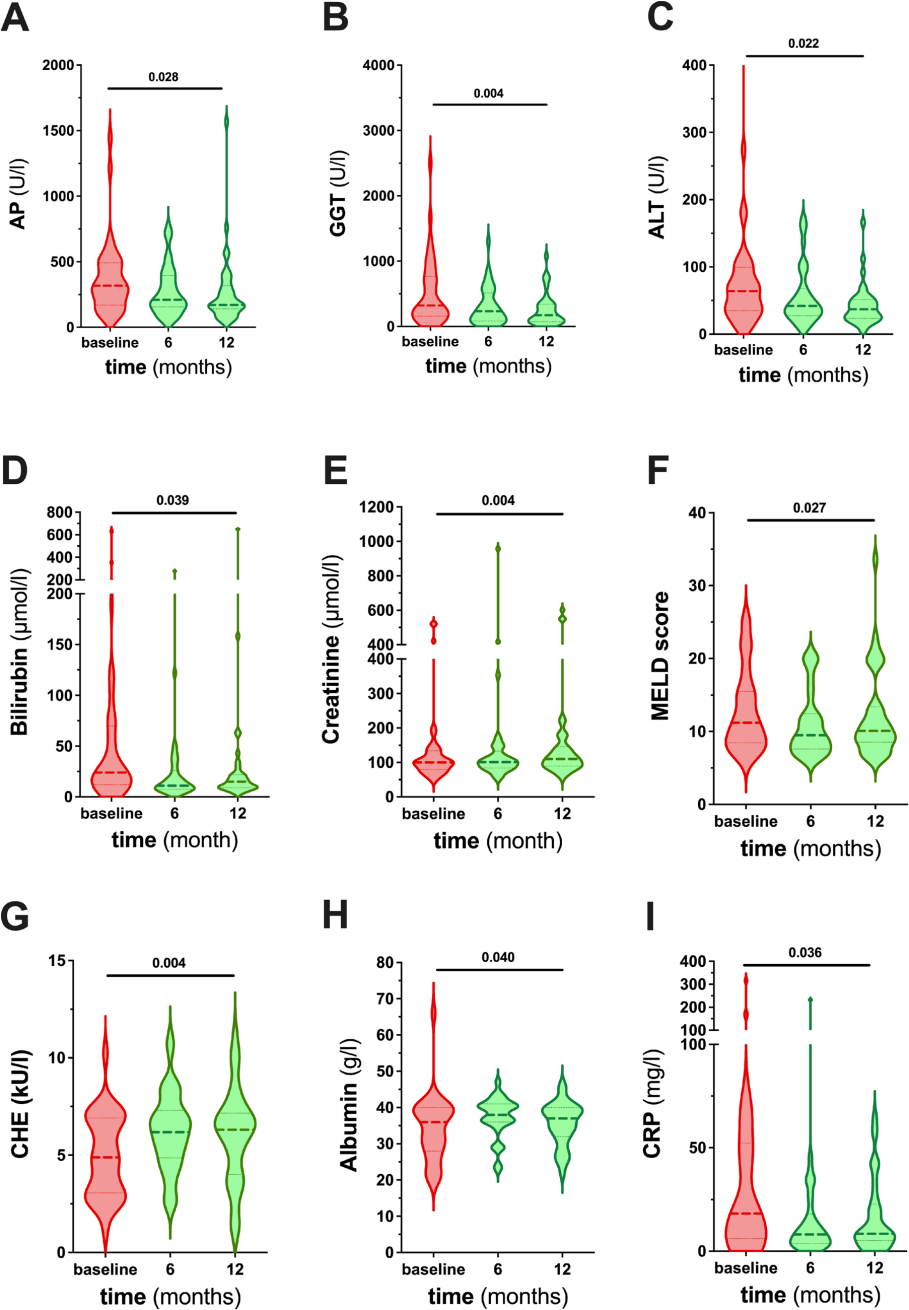
Changes in laboratory indicators of cholestasis, graft function and inflammation. Laboratory indicators of cholestasis (**A**–**D**), graft function (**G**–**H**) and inflammation (**I**) are shown as violin plots for baseline (before PTBD insertion) and follow-up (6 and 12 months following PTBD insertion) for all patients with a complete follow-up (n = 37). Values are presented as median (25% to 75% IQR). One-way ANOVA or Friedmann test were used accordingly. p-values < 0.05 were considered significant. *ALT* alanine aminotransferase, *AP* alkaline phosphatase, *CHE* cholinesterase, *CRP* C-reactive protein, *GGT* gamma glutamyl transferase, *IQR* interquartile range, *MELD* model of end stage liver disease, *PTBD* percutaneous transhepatic biliary drainage

Only patients with a complete biochemical follow-up over twelve months were taken into account in order to perform statistical tests accordingly (n = 37) (Supp. Table 1). Cholestasis parameters, such as alkaline phosphatase (AP), gamma glutamyl transferase (GGT), bilirubin as well as the C-reactive protein (CRP) were elevated at baseline representing cholestasis and cholangitis. Cholestasis was reduced significantly six and twelve months after PTBD admission (e.g. GGT: baseline: 322 (157–767) U/l vs 12 months: 172 (73–340) U/l, p = 0.004) (Fig. 2A–D). The concentration of alanine aminotransferase (ALT) and aspartate aminotransferase (AST) representing liver injury were decreased following PTBD (Fig. 2C, Supp. Fig. 2A). Albumin serum concentration and cholinesterase (CHE), representing liver synthesis performance, increased significantly (CHE: baseline: 4.89 (3.06–6.91) kU/l vs 12 months: 6.31 (4.02–7.16) kU/l, p = 0.004) (Fig. 2 G,H). The commonly used surrogate for liver graft dysfunction, the MELD score, was significantly reduced six and twelve months after PTBD admission (baseline: 11 (9–16) vs 12 months: 10 (9–13), p = 0.027) (Fig. 2F). Of note, bilirubin (Fig. 2D) and creatinine as parameters of the MELD score (Fig. 2E) decreased, while INR remained unchanged (Supp. Fig. 2B).

CRP representing inflammation was also significantly reduced during the follow-up (baseline: 18 (6–52) mg/l vs 12 months: 8 (5–23) mg/l, p = 0.036), however white blood cell count remained unchanged (Fig. 2I, Supp. Fig. 2C).

Laboratory indicators of cholestasis, inflammation and graft function are shown for baseline (PTBD insertion) and follow-up (6 and 12 months) for all patients with a complete follow-up (n = 37). Values are presented as median (25 % to 75 % IQR). p-values < 0.05 are considered significant.

### Biliary complications, need for surgical biliary revision and liver re-transplantation following PTBD

Prior to PTBD insertion biliary complications, such as cholangitis (76.4%) and cholangiosepsis (26.8%) were common and a surgical biliary revision was needed in 12 (21.4%) patients (Table 1).

**Fig. 3:**
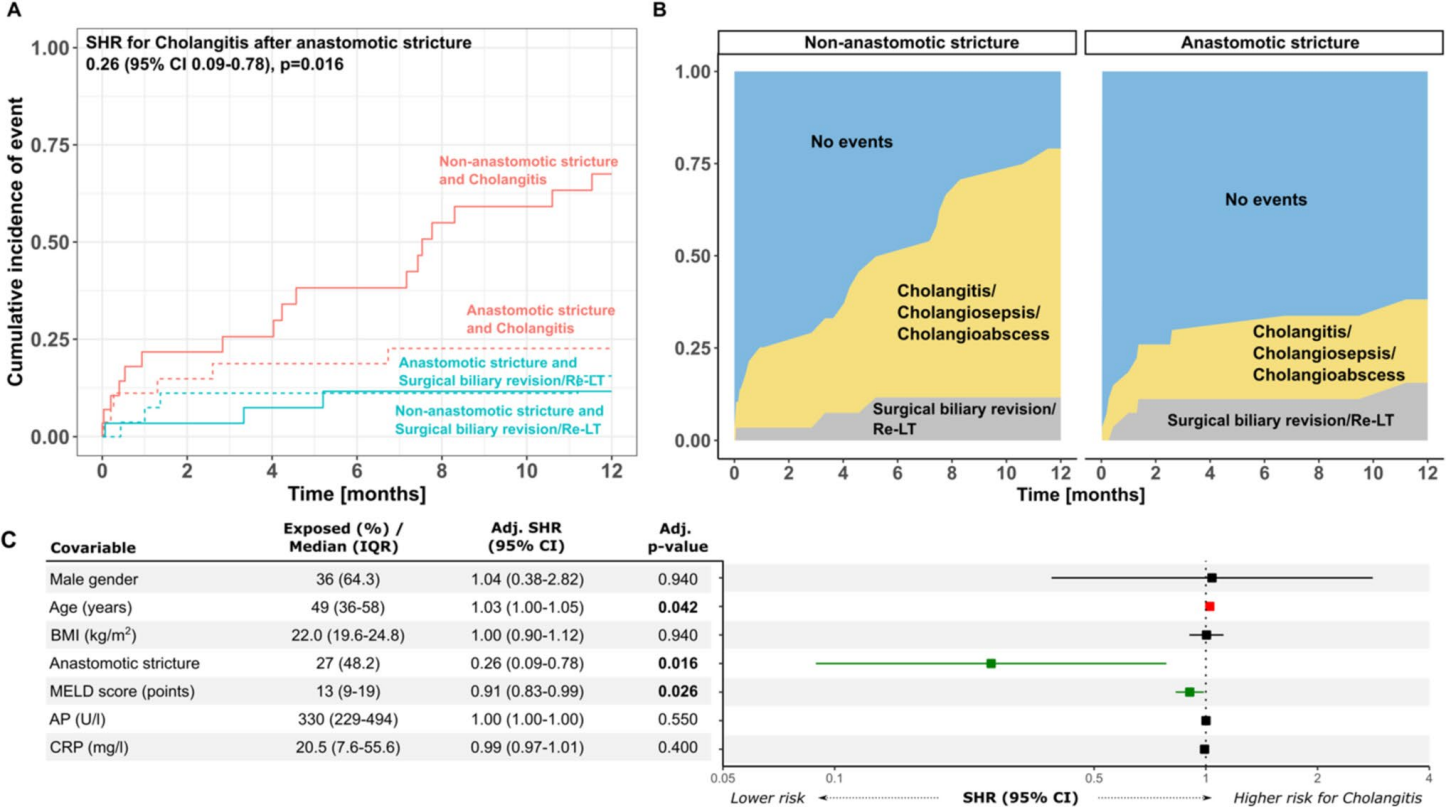
Incidence of biliary complications, surgical biliary revision and liver re-transplantation during 12 months follow up after PTBD**. A** cumulative incidence function for biliary complications and need for surgical re-intervention or re-LT (competing risk) stratified by biliary stricture type (anastomotic- vs. non-anastomotic stricture) (**A**) and a multistate comparison of the cumulative incidence of biliary complications and need for surgical re-intervention or re-LT (**B**) are shown for a one year follow up demonstrating a lower incidence of biliary complications in patients with an anastomotic stricture. (**C**) Multivariable competing risk regression with subhazard ratios (SHR) and 95% CIs is shown as a forest plot with corresponding table. p-values < 0.05 were considered significant. *AP* alkaline phosphatase, *AS* anastomotic stricture, *BMI* body mass index, *CRP* C-reactive protein, *IQR* interquartile range, *LT* liver transplantation, *MELD* model of end stage liver disease, *NAS* non-anastomotic stricture, *PTBD* percutaneous transhepatic biliary drainage, *SHR* subhazard ratio

Following PTBD insertion, biliary complications occurred in a total of 23 (41.1%) patients after a median of 7.3 (IQR 1.2–22.6) months during a one-year follow-up. Patients receiving a PTBD for AS were significantly less likely to develop biliary complications than patients with a NAS (cumulative incidence: AS 22.6% vs NAS 67.5%) (Fig. 3A, B). A surgical re-intervention or re-LT was performed in a total of seven patients (12.5%) during 12-month follow-up. Here, the nature of biliary stricture did not affect if a surgical re-intervention or liver re-LT needed to be performed (Fig. 3A, B). In a multivariate competing risk regression model (Fig. 3C), AS stenosis was the strongest predictor of subsequent biliary complications (adjusted sub-Hazard Ratio (SHR): 0.26, 95% CI: 0.09–0.78, p = 0.016). Patient age increased the risk for cholangitis (SHR 1.03, 95% CI: 1.00–1.05, p = 0.042), whereas a higher initial MELD score was associated with a numerically lower risk for cholangitis (SHR 0.91, 95% CI: 0.83–0.99, p = 0.026). Neither patient gender and body mass index (BMI) nor baseline AP and CRP concentration had an influence on the incidence of consecutive biliary complications following PTBD (Fig. 3C).

Long-term analysis within a follow-up period of 60 months since first PTBD insertion, confirmed the main finding of AS being the major predictor of reduced biliary complications following PTBD (Supp. Fig. 3A–C).

### Short- and long-term survival in patients receiving PTBD following LT

**Fig. 4:**
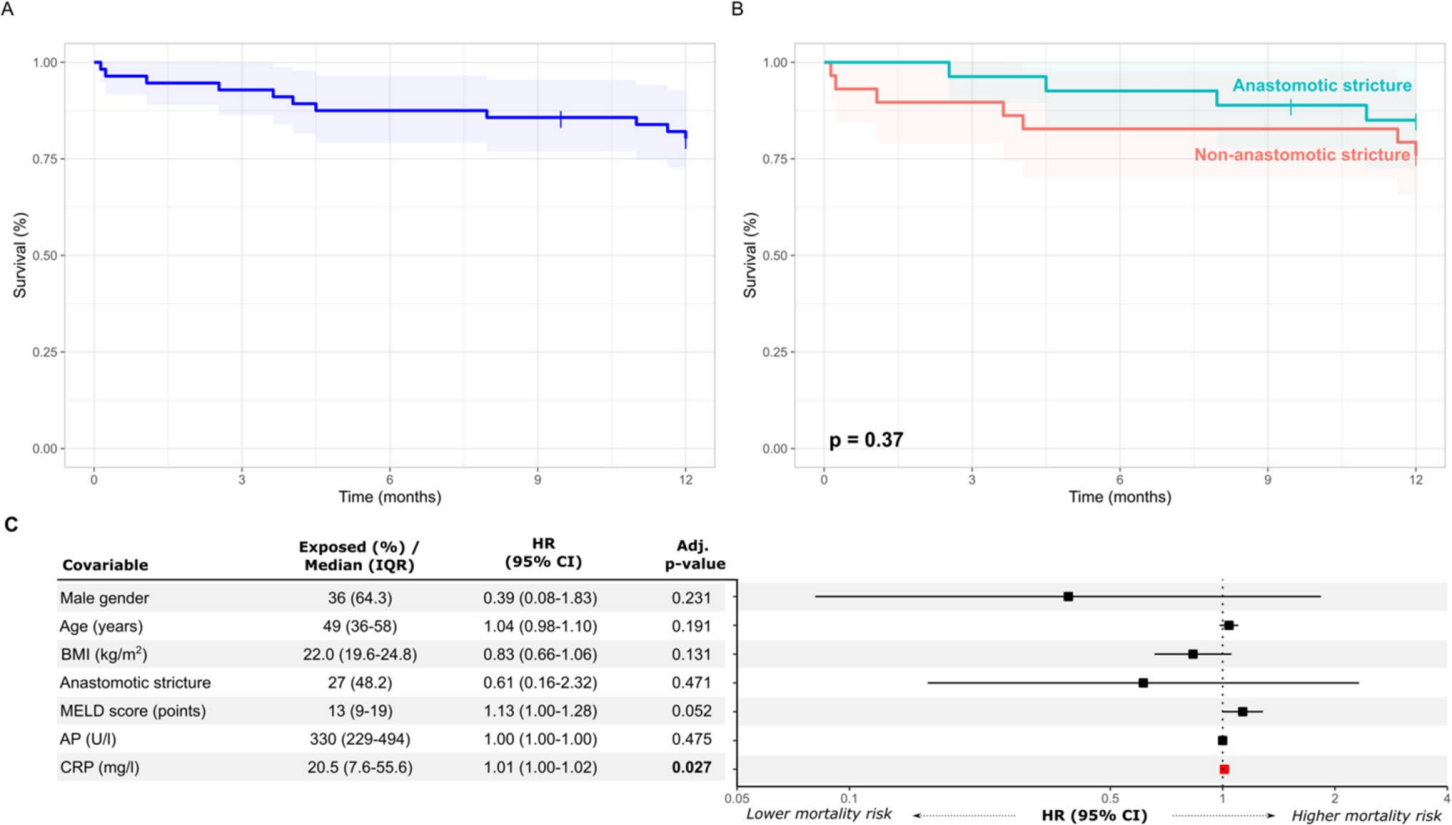
12-month survival following PTBD**.** Survival after initial PTBD insertion is shown as Kaplan–Meier graphs for all patients (**A**) or patients stratified by biliary stricture type (**B**) for a one-year follow-up. (**C**) Multivariate survival cox regression with hazard ratios (HR) and 95% CIs is shown as a forest plot with corresponding table. p-values < 0.05 are considered significant. p-values < 0.05 were considered significant. *AP* alkaline phosphatase, *AS* anastomotic stricture, *BMI* body mass index, *CRP* C-reactive protein, *HR* hazard ratio, *IQR* interquartile range, *LT* liver transplantation, *MELD* model of end stage liver disease, *NAS* non-anastomotic stricture, *PTBD* percutaneous transhepatic biliary drainage

12-month overall survival was 80% (95% CI: 70–91%) (Fig. 4A). Different potential predictors of 12 months survival were analyzed by multivariate cox-proportional hazard regression (Fig. 4B, C). Although AS was numerically associated with a lower SHR for 12-months mortality, this was not statistically significant. Of all investigated parameters, only higher CRP at baseline was associated with increased mortality at 12 months following PTBD (SHR 1.01, 95% CI: 1.00–1.02, p = 0.027). Long-term survival up to 60 months was 57% (Supp. Fig.4A) and was numerically higher in patients with AS compared to those with NAS (70% vs 46%, p = 0.11) (Supp. Fig. 4B). Again, higher baseline CRP concentration and also lower BMI was associated with higher mortality at 60 months (Supp. Fig. 4C).

**Fig. 5:**
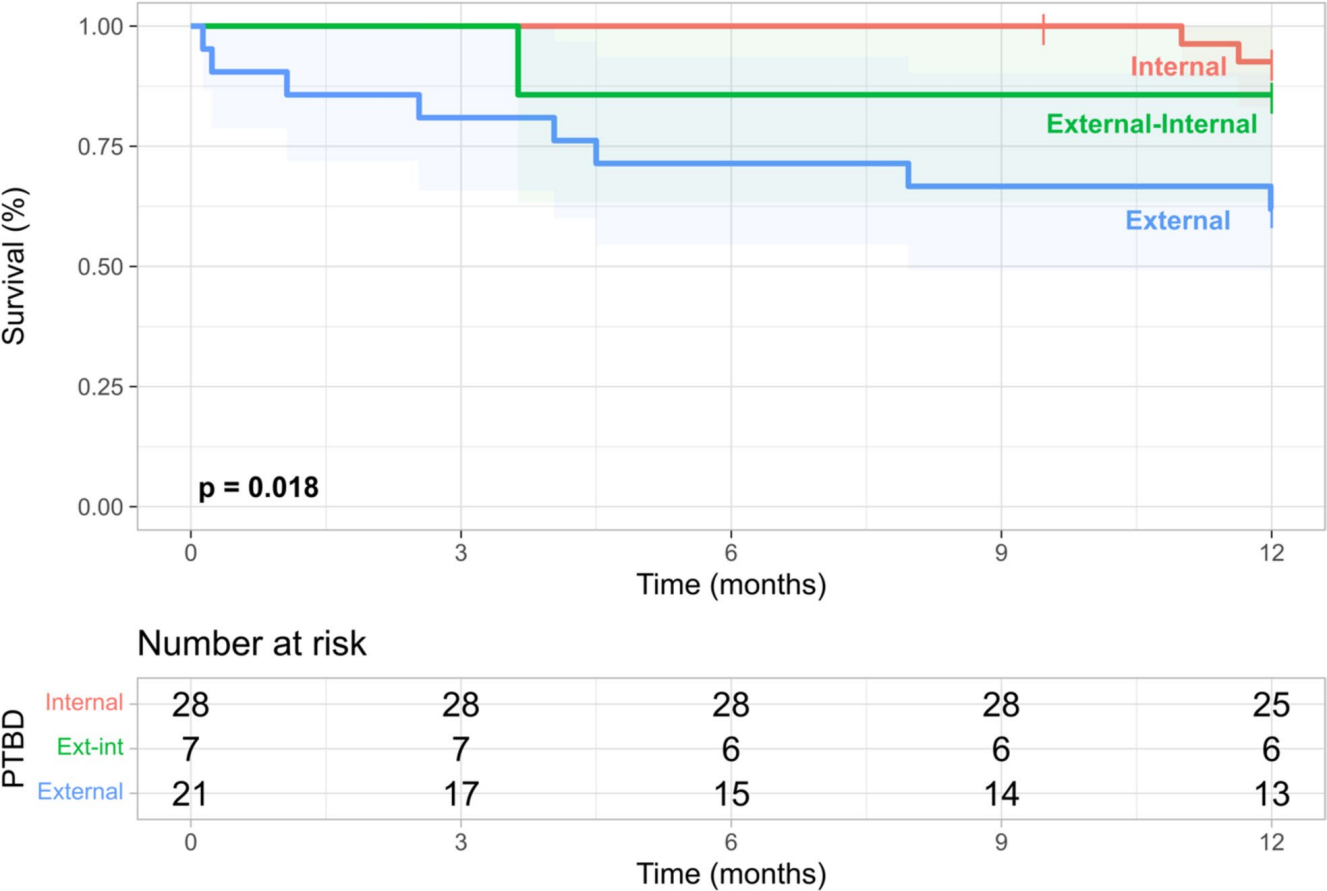
Influence of PTBD internalization on 12-month survival. Survival after initial PTBD insertion is shown as Kaplan–Meier graphs for patients stratified by ability to internalize PTBD on first attempt (Internal) vs. on subsequent attempts (External-Internal) vs. never (External) for a one-year follow-up. *PTBD* Percutaneous transhepatic biliary drainage

Interestingly, patients, in which an internal PTBD drainage into the small intestine was achieved at first attempt had a significantly better 12 months survival than patients, in which internalization could either never or only on consecutive PTBD attempts be achieved (Fig. 5, p = 0.018) 25 (93%) patients with an initially internal placed PTBD survived after 12 months, whereas ‘only’ 19 (68%) patients with an initially external PTBD survived during the same follow-up. However, long term follow-up to 60 months did no longer show significant survival differences dependent on PTBD placement strategy (Supp. Fig. 5).

## Discussion

In this retrospective, single-center study, short and long-term clinical outcomes and outcome predictors were systematically analyzed in 56 LT adults who received at least one PTBD procedure for cholestasis without alternative biliary access. PTBD procedures had a tolerable safety profile and a high technical success rate. Multiple laboratory indicators of cholestasis, graft function and inflammation improved during a 12-month follow-up. Incidence of subsequent biliary complications was highly dependent on the nature of biliary strictures, as AS were the strongest predictor of reduced biliary complications both in 12- and 60 months follow-up. Need for surgical biliary revision or re-LT remained below 15% and were independent of the type of biliary stricture. Patients, in which an internal PTBD drainage into the small intestine was achieved at first attempt had a significantly better 12-month survival than patients, in which internalization could either never or only on consecutive PTBD attempts be achieved. Patients with AS had a numerically higher long-term survival at 60 months than patients with NAS and both higher CRP and lower BMI at baseline were associated with inferior short- and long-term survival.

Although ERC as a standard biliary access may not be feasible in a significant proportion of patients with biliary strictures following LT [2, 3, 7], therefore then requiring alternative access by PTBD, only few and rather small studies so far systematically investigated outcome in these patients. In one of the first descriptions of 27 patients receiving PTBD after LT, only five patients survived without re-LT during a five year follow-up [15]. Later series from other centers reported higher success rates without surgical biliary revision or re-LT of 60% [16], 51% [11], and 75% [17], respectively. In the present series need for surgical biliary revision and re-LT remained below 15% indicating a high success of a PTBD strategy in these patients.

Previous studies mainly described therapeutic success primarily as resolution of strictures assessed by the interventionalist [7, 11, 15, 16] without systematically reporting incidence of biliary complications (cholangitis/cholangiosepsis/cholangioabscess) following PTBD. In contrast, this study therefore focused on these clinical objective endpoints analyzed in competing-risk models together with the need for surgical biliary revision or re-LT. In fact, a significant proportion of patients experiences recurrent biliary complications despite PTBD insertion during both short- and long-term follow-up. We chose 12- and 60-month follow-up to indicate short- and long-term follow-up, rather than timepoints in between (e.g. 36 months) to clearly differ between these time periods.

Of note, the frequency of biliary complications following PTBD was heterogenous and highly dependent on the nature of biliary stricture addressed by PTBD. Patients with AS treated by PTBD were much less likely to develop subsequent biliary complications than patients with NAS. Only few studies identified potential outcome predictors in patients with biliary strictures following LT treated by PTBD [7, 11] while others did not specifically describe such predictors in PTBD treated patients [7] or could not identify significant predictors at all [17]. Sung et al. identified older age at transplant and singular strictures as factors predicting treatment success [11]. While older age was not significantly associated with biliary complications in this analysis, AS, as a more isolated stricture in contrast to the multiple NAS, was strongly associated with reduced complications. In a more recent analysis of combined AS and NAS treated with combined ERC and PTBD, a baseline CRP above 8 mg/l and a BMI below 21 kg/m^2^ at time of transplantation were both associated with subsequent treatment failure [7]. In the present analysis incidence of biliary complications despite PTBD was independent of CRP and BMI, but both higher CRP and lower BMI were associated with increased mortality following PTBD. Of interest, failure to achieve internal biliary drainage into the small intestine at first PTBD insertion was also significantly associated with increased short-term mortality. This has not yet been described as a prognostic parameter associated in patients with PTBD following LT. It however appears understandable that strictures, which cannot initially be interventional bridged by PTBD, might represent a phenotypical”difficult to resolve” subcohort, therefore predisposing to persisting cholestasis and its associated adverse outcomes.

This study has some limitations, mainly its retrospective character and the single center setting limiting generalizability of the data. In this study a significant number of patients received a LT due to PSC, with a high variability of cholangitis episodes prior to LT, which might also influence probability of cholangitis episodes occurring after LT. The results, suggesting reduced risk of biliary complications in patients with AS and increased mortality in patients with higher CRP, lower BMI and failure of initial PTBD internalization, are provocative but should be interpreted as rather hypothesis-generating as they need further evaluation in larger and preferably prospective multicenter studies. Strengths of this study are the relatively large patient number included, the long follow-up and the robust statistical methods applied, using both multivariate competing risk and cox-proportional hazard modelling, to analyze multiple, clinical important endpoints and potential prediction variables of these outcomes.

## Conclusions

In summary, PTBD in LT adults without any alternative biliary access had a high technical success rate, a tolerable safety profile and were associated with improved laboratory indicators of cholestasis, graft function and inflammation. Incidence of subsequent biliary complications was highly dependent on the nature of present biliary strictures as AS were associated with a lower risk of such complications. Need for surgical biliary revision and re-LT was below 15% and independent of stricture type. Increased mortality was found in patients with higher CRP and lower BMI at baseline, and failure of initial PTBD internalization.

## Supplementary Information

Below is the link to the electronic supplementary material.

Supplementary file1 (TIFF 49219 KB) PTBD demission and need for reinsertion. Percentage of total patients with PTBD demission (A) and without PTBD reinsertion (C) or percentage of patients stratified by biliary stricture type (anastomotic- vs. non-anastomotic stricture) with PTBD demission (B) and without PTBD reinsertion (D) are shown as Kaplan-Meier graphs for a two-year follow-up. p-values < 0.05 are considered significant. PTBD percutaneous transhepatic biliary drainage

Supplementary file2 (TIFF 786 KB) Additional laboratory indicators of liver injury, graft function and inflammation. AST (A), INR (B) and white cell count (C) are shown as violin plots for baseline (before PTBD insertion) and follow-up (6 and 12 months following PTBD insertion) for all patients with a complete follow-up (n = 37). One-way ANOVA or Friedmann test were used accordingly. Values are presented as median (25 % to 75 % IQR). p-values < 0.05 are considered significant. AST aspartate aminotransferase, INR international normalized ratio, IQR interquartile range, PTBD percutaneous transhepatic biliary drainage

Supplementary file3 (TIFF 40211 KB) Incidence of biliary complications, surgical biliary revision and liver re-transplantation during 60 months follow up after PTBD. A cumulative incidence function for biliary complications and need for surgical re-intervention or re-LT (competing risk) stratified by biliary stricture type (anastomotic- vs. non-anastomotic stricture) (A) and a multistate comparison of the cumulative incidence of biliary complications and need for surgical re-intervention or re-LT (B) are shown for a five year follow up demonstrating a lower incidence of biliary complications in patients with a anastomotic stricture (C) Multivariable competing risk regression with subhazard ratios (SHR) and 95% CIs is shown as a forest plot with corresponding table. p-values < 0.05 were considered significant. AP alkaline phosphatase, AS anastomotic stricture, BMI body mass index, CRP C-reactive protein, IQR interquartile range, LT liver transplantation, MELD model of end stage liver disease, NAS non-anastomotic stricture, PTBD percutaneous transhepatic biliary drainage, SHR subhazard ratio

Supplementary file4 (TIFF 40211 KB) 60-month survival following PTBD. Survival after initial PTBD insertion is shown as Kaplan-Meier graphs for all patients (A) or patients stratified by biliary stricture type (B) for a five-year follow-up. (C) Multivariate survival cox regression with hazard ratios (HR) and 95% CIs is shown as a forest plot with corresponding table. p-values < 0.05 are considered significant. p-values < 0.05 were considered significant. AP alkaline phosphatase, AS anastomotic stricture, BMI body mass index, CRP C-reactive protein, HR hazard ratio, IQR interquartile range, LT liver transplantation, MELD model of end stage liver disease, NAS non-anastomotic stricture, PTBD percutaneous transhepatic biliary drainage

Supplementary file5 (TIFF 12305 KB) Influence of PTBD internalization on 60-month survival. Survival after initial PTBD insertion is shown as Kaplan-Meier graphs for patients stratified by ability to internalize PTBD on first attempt (Internal) vs. on subsequent attempts (External-Internal) vs. never (External) for a five-year follow-up. PTBD percutaneous transhepatic biliary drainage

Supplementary file6 (DOCX 21 KB)

## Data Availability

The data underlying this article will be shared on reasonable request to the corresponding author.

## Abbreviations

AP: Alkaline phosphatase
AS: Anastomotic stricture
AST: Aspartate aminotransferase
CRP: C-reactive protein
ERC: Endoscopic retrograde cholangioscopy
GGT: Gamma glutamyl transferase
LT: Liver transplantation
MELD: Model for the end stage of liver disease
NAS: Non-anastomotic stricture
PTBD: Percutaneous transhepatic biliary drainage

## References

[1] Magro B, Tacelli M, Mazzola A, Conti F, Celsa C. Biliary complications after liver transplantation: current perspectives and future strategies. Hepatobiliary Surg Nutr. 2021;10(1):76-92. 10.21037/hbsn.2019.09.01. 33575291 10.21037/hbsn.2019.09.01 PMC7867735

[2] Akamatsu N, Sugawara Y, Hashimoto D. Biliary reconstruction, its complications and management of biliary complications after adult liver transplantation: a systematic review of the incidence, risk factors and outcome. Transpl Int. 2011;24(4):379-92. 10.1111/j.1432-2277.2010.01202.x. 21143651 10.1111/j.1432-2277.2010.01202.x

[3] Roos FJM, Poley JW, Polak WG, Metselaar HJ. Biliary complications after liver transplantation; recent developments in etiology, diagnosis and endoscopic treatment. Best Pract Res Clin Gastroenterol. 2017;31(2):227-35. 10.1016/j.bpg.2017.04.002. 28624111 10.1016/j.bpg.2017.04.002

[4] Lee HW, Shah NH, Lee SK. An Update on Endoscopic Management of Post-Liver Transplant Biliary Complications. Clin Endosc. 2017;50(5):451-63. 10.5946/ce.2016.139. 28415168 10.5946/ce.2016.139 PMC5642064

[5] Kochhar G, Parungao JM, Hanouneh IA, Parsi MA. Biliary complications following liver transplantation. World J Gastroenterol. 2013;19(19):2841-6. 10.3748/wjg.v19.i19.2841. 23704818 10.3748/wjg.v19.i19.2841 PMC3660810

[6] Verdonk RC, Buis CI, Porte RJ, Haagsma EB. Biliary complications after liver transplantation: a review. Scand J Gastroenterol Suppl. 2006(243):89-101. 10.1080/00365520600664375. 10.1080/00365520600664375 16782628

[7] Heinemann M, Tafrishi B, Pischke S, Fischer L, Rösch T, Lohse AW, et al. Endoscopic retrograde cholangiography and percutaneous transhepatic cholangiodrainage in biliary strictures after liver transplantation: Long-term outcome predictors and influence on patient survival. Liver Int. 2019;39(6):1155-64. 10.1111/liv.13995. 30367552 10.1111/liv.13995

[8] Sharma S, Gurakar A, Jabbour N. Biliary strictures following liver transplantation: past, present and preventive strategies. Liver Transpl. 2008;14(6):759-69. 10.1002/lt.21509. 18508368 10.1002/lt.21509

[9] Verdonk RC, Buis CI, Porte RJ, van der Jagt EJ, Limburg AJ, van den Berg AP, et al. Anastomotic biliary strictures after liver transplantation: causes and consequences. Liver Transpl. 2006;12(5):726-35. 10.1002/lt.20714. 16628689 10.1002/lt.20714

[10] Hildebrand T, Pannicke N, Dechene A, Gotthardt DN, Kirchner G, Reiter FP, et al. Biliary strictures and recurrence after liver transplantation for primary sclerosing cholangitis: A retrospective multicenter analysis. Liver Transpl. 2016;22(1):42-52. 10.1002/lt.24350. 26438008 10.1002/lt.24350

[11] Sung RS, Campbell DA, Jr., Rudich SM, Punch JD, Shieck VL, Armstrong JM, et al. Long-term follow-up of percutaneous transhepatic balloon cholangioplasty in the management of biliary strictures after liver transplantation. Transplantation. 2004;77(1):110-5. 10.1097/01.Tp.0000101518.19849.C8. 14724444 10.1097/01.TP.0000101518.19849.C8

[12] Kiriyama S, Kozaka K, Takada T, Strasberg SM, Pitt HA, Gabata T, et al. Tokyo Guidelines 2018: diagnostic criteria and severity grading of acute cholangitis (with videos). J Hepatobiliary Pancreat Sci. 2018;25(1):17-30. 10.1002/jhbp.512. 29032610 10.1002/jhbp.512

[13] Singer M, Deutschman CS, Seymour CW, Shankar-Hari M, Annane D, Bauer M, et al. The Third International Consensus Definitions for Sepsis and Septic Shock (Sepsis-3). Jama. 2016;315(8):801-10. 10.1001/jama.2016.0287. 26903338 10.1001/jama.2016.0287 PMC4968574

[14] Kamath PS, Wiesner RH, Malinchoc M, Kremers W, Therneau TM, Kosberg CL, et al. A model to predict survival in patients with end-stage liver disease. Hepatology. 2001;33(2):464-70. 10.1053/jhep.2001.22172. 11172350 10.1053/jhep.2001.22172

[15] Zajko AB, Bron KM, Campbell WL, Behal R, Van Thiel DH, Starzl TE. Percutaneous transhepatic cholangiography and biliary drainage after liver transplantation: a five-year experience. Gastrointest Radiol. 1987;12(2):137-43. 10.1007/bf01885124. 3549417 10.1007/BF01885124 PMC2967184

[16] Barton P, Maier A, Steininger R, Mühlbacher F, Lechner G. Biliary sludge after liver transplantation: 1. Imaging findings and efficacy of various imaging procedures. AJR Am J Roentgenol. 1995;164(4):859-64. 10.2214/ajr.164.4.7726038. 7726038 10.2214/ajr.164.4.7726038

[17] Hung HH, Chen TS, Tseng HS, Hsia CY, Liu CS, Lin HC, et al. Percutaneous transhepatic cholangiography and drainage is an effective rescue therapy for biliary complications in liver transplant recipients who fail endoscopic retrograde cholangiopancreatography. J Chin Med Assoc. 2009;72(8):395-401. 10.1016/s1726-4901(09)70395-8. 19686994 10.1016/S1726-4901(09)70395-8

