# Supplementary figures and images for: Percutaneous transhepatic biliary drainage in patients with cholestasis following liver transplantation
Source: Abdom Radiol (NY). 2024 Nov 5;50(4):1699–710. doi: 10.1007/s00261-024-04657-2 (PMC11947054; doi:10.1007/s00261-024-04657-2)

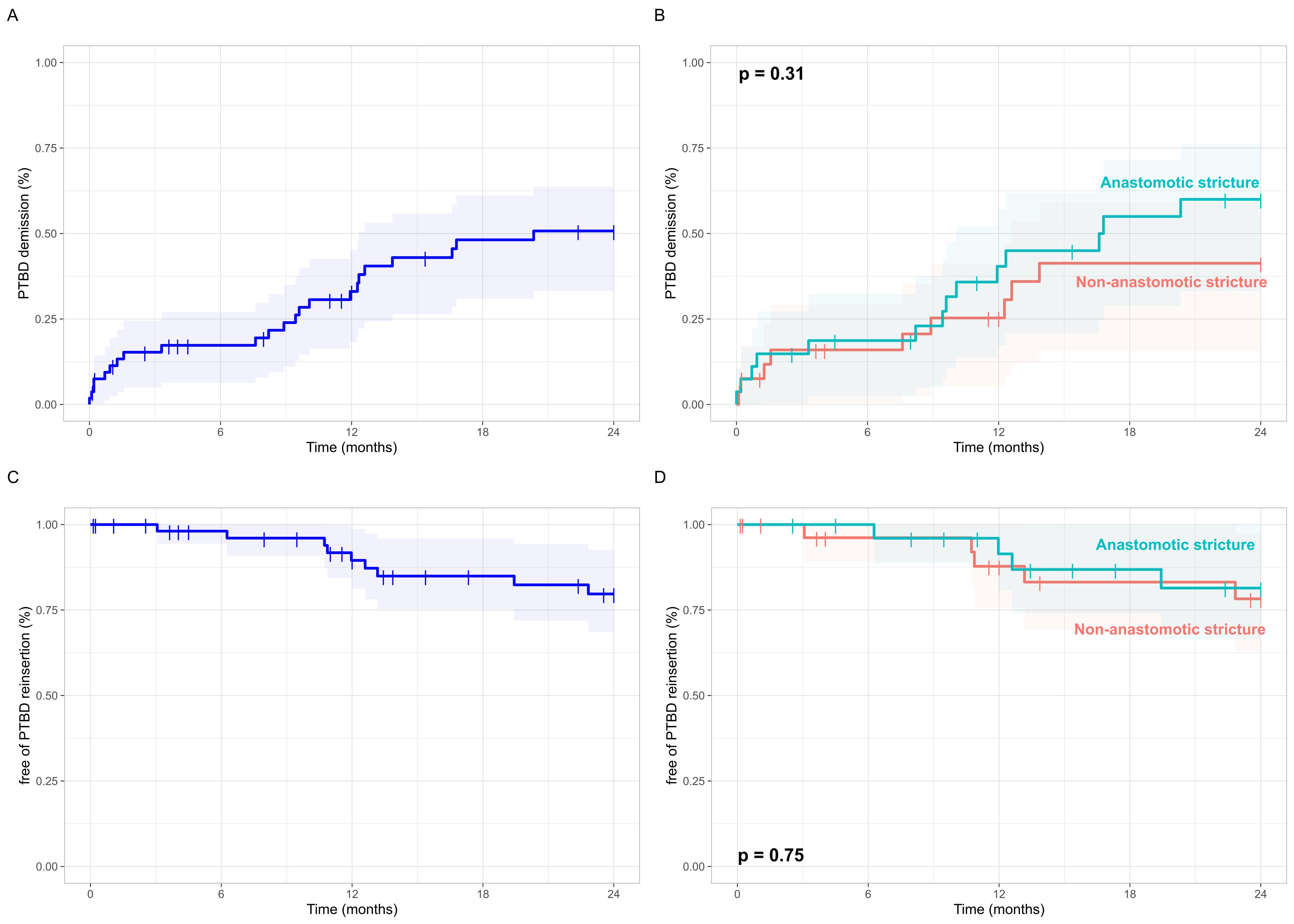

Supplement: Supplementary file 1 — Supplementary file1 (TIFF 49219 KB) PTBD demission and need for reinsertion. Percentage of total patients with PTBD demission (A) and without PTBD reinsertion (C) or percentage of patients stratified by biliary stricture type (anastomotic- vs. non-anastomotic stricture) with PTBD demission (B) and without PTBD reinsertion (D) are shown as Kaplan-Meier graphs for a two-year follow-up. p-values < 0.05 are considered significant. PTBD percutaneous transhepatic biliary drainage [file 261_2024_4657_MOESM1_ESM.tiff]

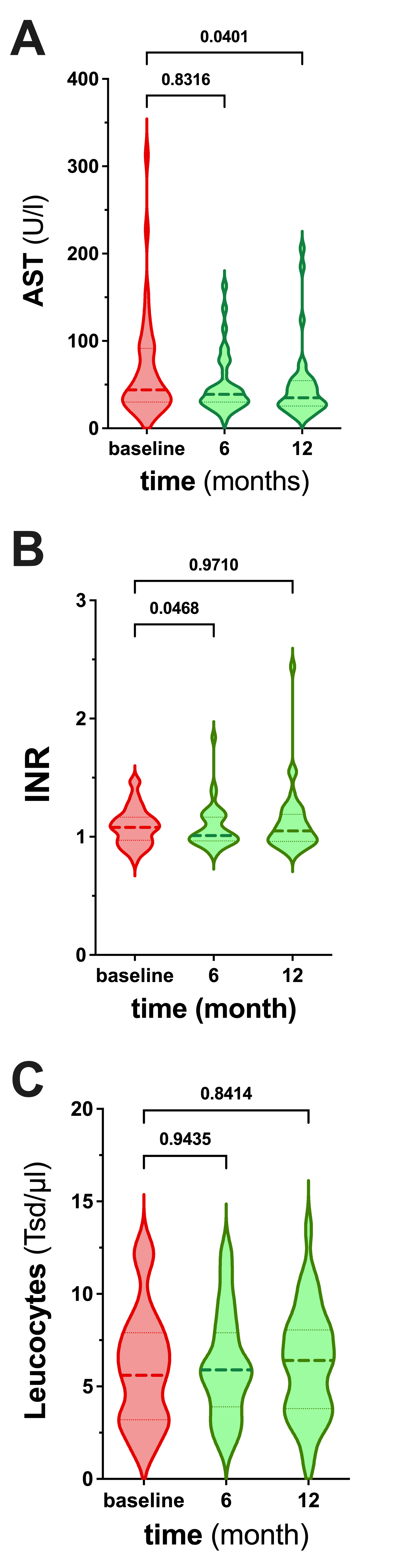

Supplement: Supplementary file 2 — Supplementary file2 (TIFF 786 KB) Additional laboratory indicators of liver injury, graft function and inflammation. AST (A), INR (B) and white cell count (C) are shown as violin plots for baseline (before PTBD insertion) and follow-up (6 and 12 months following PTBD insertion) for all patients with a complete follow-up (n = 37). One-way ANOVA or Friedmann test were used accordingly. Values are presented as median (25 % to 75 % IQR). p-values < 0.05 are considered significant. AST aspartate aminotransferase, INR international normalized ratio, IQR interquartile range, PTBD percutaneous transhepatic biliary drainage [file 261_2024_4657_MOESM2_ESM.tiff]

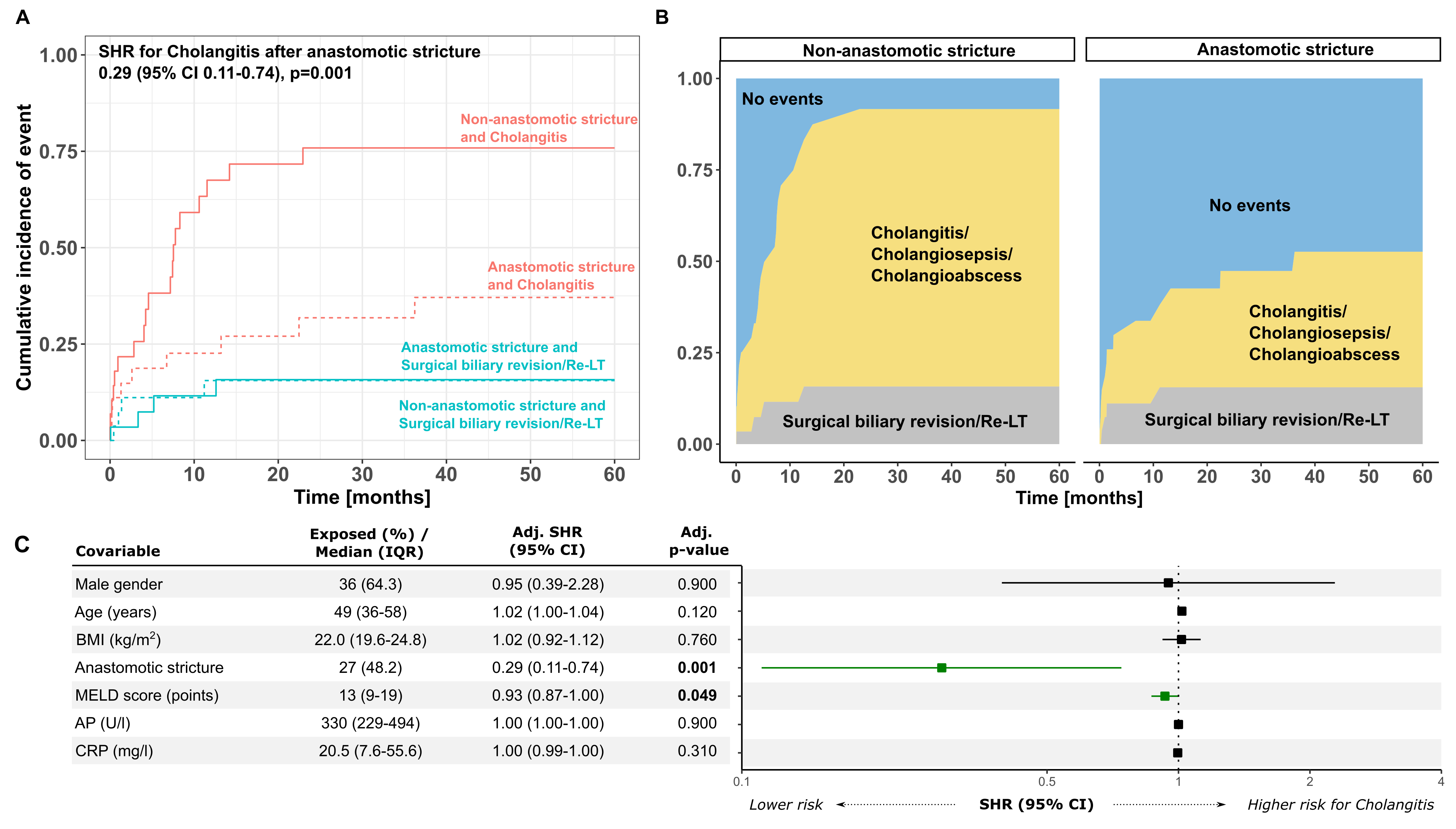

Supplement: Supplementary file 3 — Supplementary file3 (TIFF 40211 KB) Incidence of biliary complications, surgical biliary revision and liver re-transplantation during 60 months follow up after PTBD. A cumulative incidence function for biliary complications and need for surgical re-intervention or re-LT (competing risk) stratified by biliary stricture type (anastomotic- vs. non-anastomotic stricture) (A) and a multistate comparison of the cumulative incidence of biliary complications and need for surgical re-intervention or re-LT (B) are shown for a five year follow up demonstrating a lower incidence of biliary complications in patients with a anastomotic stricture (C) Multivariable competing risk regression with subhazard ratios (SHR) and 95% CIs is shown as a forest plot with corresponding table. p-values < 0.05 were considered significant. AP alkaline phosphatase, AS anastomotic stricture, BMI body mass index, CRP C-reactive protein, IQR interquartile range, LT liver transplantation, MELD model of end stage liver disease, NAS non-anastomotic stricture, PTBD percutaneous transhepatic biliary drainage, SHR subhazard ratio [file 261_2024_4657_MOESM3_ESM.tiff]

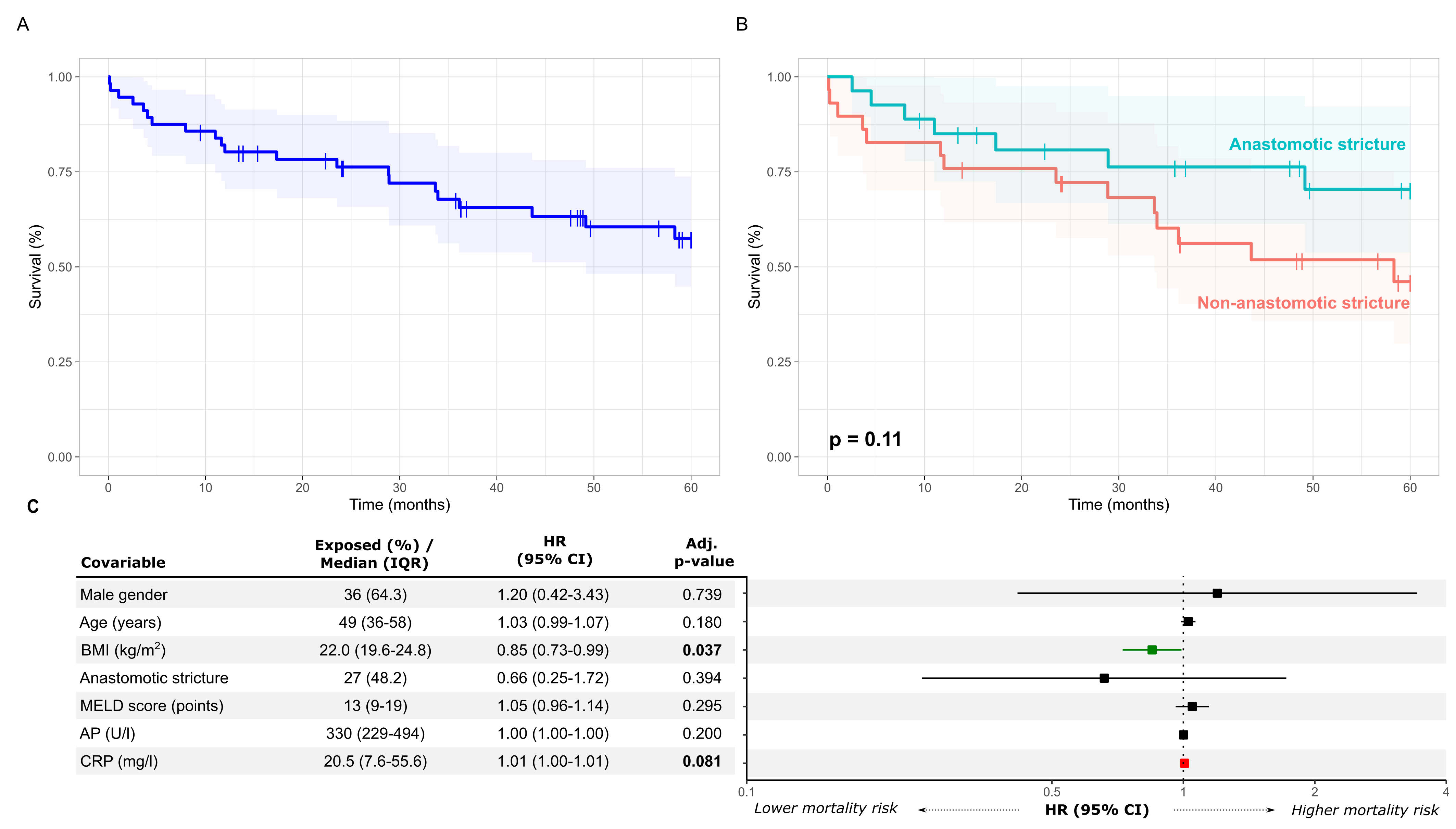

Supplement: Supplementary file 4 — Supplementary file4 (TIFF 40211 KB) 60-month survival following PTBD. Survival after initial PTBD insertion is shown as Kaplan-Meier graphs for all patients (A) or patients stratified by biliary stricture type (B) for a five-year follow-up. (C) Multivariate survival cox regression with hazard ratios (HR) and 95% CIs is shown as a forest plot with corresponding table. p-values < 0.05 are considered significant. p-values < 0.05 were considered significant. AP alkaline phosphatase, AS anastomotic stricture, BMI body mass index, CRP C-reactive protein, HR hazard ratio, IQR interquartile range, LT liver transplantation, MELD model of end stage liver disease, NAS non-anastomotic stricture, PTBD percutaneous transhepatic biliary drainage [file 261_2024_4657_MOESM4_ESM.tiff]

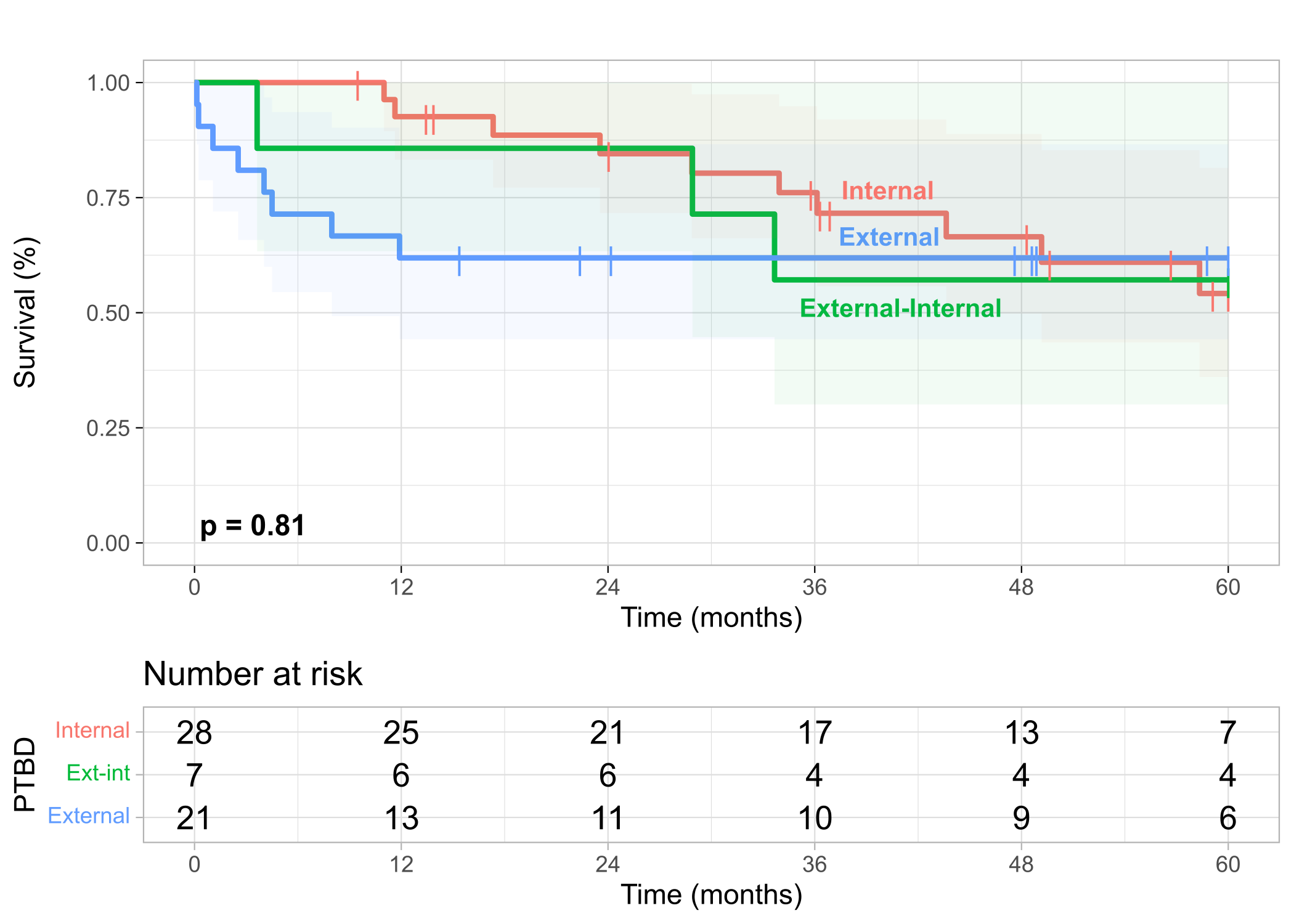

Supplement: Supplementary file 5 — Supplementary file5 (TIFF 12305 KB) Influence of PTBD internalization on 60-month survival. Survival after initial PTBD insertion is shown as Kaplan-Meier graphs for patients stratified by ability to internalize PTBD on first attempt (Internal) vs. on subsequent attempts (External-Internal) vs. never (External) for a five-year follow-up. PTBD percutaneous transhepatic biliary drainage [file 261_2024_4657_MOESM5_ESM.tiff]
