# Supplementary material for: Percutaneous transhepatic biliary drainage in patients with cholestasis following liver transplantation
Source: Abdom Radiol (NY). 2024 Nov 5;50(4):1699–710. doi: 10.1007/s00261-024-04657-2 (PMC11947054; doi:10.1007/s00261-024-04657-2)
Supplement: Supplementary file 6 — Supplementary file6 (DOCX 21 KB) [file 261_2024_4657_MOESM6_ESM.docx]

| **Suppl. Table 1:** Biochemical characteristics at PTBD insertion and follow-up (6 and 12 months). | | | | |
| --- | --- | --- | --- | --- |
| **Category** | **Median (IQR)/No (%)** | | | |
|  | (n=37) | | | |
|  | **baseline** | **6 months after PTBD** | **12 months after PTBD** | **p-value** |
| AP - U/l | 318 (170-493) | 211 (156-395) | 170 (140-318) | **0.028** |
| GGT - U/l | 322 (157-767) | 233 (83-511) | 172 (73-340) | **0.004** |
| AST - U/l | 44 (30-92) | 39 (30-56) | 35 (26-55) | 0.060 |
| ALT - U/l | 64 (35-100) | 42 (28-68) | 37 (24-52) | **0.022** |
| Bilirubin total - µmol/l | 24 (12-70) | 11 (8-26) | 15 (9-22) | **0.039** |
| INR | 1.08 (0.97-1.17) | 1.01 (0.97-1.17) | 1.05 (0.96-1.19) | 0.060 |
| Creatinine - µmol/l | 100 (80-134) | 101 (84-133) | 110 (90-146) | **0.004** |
| Sodium – mmol/l | 139 (135-141) | 139 (137-141) | 139 (136-140) | 0.760 |
| MELD | 11 (9-16) | 10 (8-13) | 10 (9-13) | **0.027** |
| CHE – kU/l | 4.89 (3.06-6.91) | 6.18 (4.86-7.30) | 6.31 (4.02-7.16) | **0.004** |
| Albumin – g/l | 36 (28-40) | 38 (36-41) | 37 (32-40) | **0.040** |
| CRP – mg/l | 18.2 (6.05-52.3) | 8.1 (3.7-18) | 8.4 (5.2-22.8) | **0.036** |
| Leucocytes – Tsd/µl | 5.6 (3.2-7.9) | 5.9 (3.9-7.9) | 6.4 (3.8-8.1) | 0.886 |

ABBREVIATIONS:

ALT – Alanine aminotransferase, AP – Alkaline phosphatase, AST – Aspartate aminotransferase, CHE - Cholinesterase, CRP – C-reactive protein, GGT – Gamma glutamyl transferase, INR – International normalized ratio, MELD – Model of end stage liver disease, PTBD - Percutaneous transhepatic biliary drainage
